# RETRACTION: The Effect of Placing Drains and No Drains After Caesarean Section in Obese Patients on Patients' Post‐Operative Wound Complications: A Meta‐Analysis

**DOI:** 10.1111/iwj.70393

**Published:** 2025-03-21

## Abstract

**RETRACTION:** ZhengY. , ZhaoF. , NingF. , and LiN. , “The Effect of Placing Drains and No Drains After Caesarean Section in Obese Patients on Patients' Post‐Operative Wound Complications: A Meta‐Analysis,” International Wound Journal 21, no. 2 (2024): e14576, 10.1111/iwj.14576.PMC1083191542053017

The above article, published online on 01 February 2024, in Wiley Online Library (http://onlinelibrary.wiley.com/), has been retracted by agreement between the journal Editor in Chief, Professor Keith Harding; and John Wiley & Sons Ltd. Following an investigation by the publisher, all parties have concluded that this article was accepted solely on the basis of a compromised peer review process. The editors have therefore decided to retract the article. The authors did not respond to our notice regarding the retraction.

**RETRACTION:** Y. Zheng , F. Zhao , F. Ning , and N. Li , “The Effect of Placing Drains and No Drains After Caesarean Section in Obese Patients on Patients' Post‐Operative Wound Complications: A Meta‐Analysis,” International Wound Journal 21, no. 2 (2024): e14576, 10.1111/iwj.14576.PMC1083191542053017

The above article, published online on 01 February 2024, in Wiley Online Library (http://onlinelibrary.wiley.com/), has been retracted by agreement between the journal Editor in Chief, Professor Keith Harding; and John Wiley & Sons Ltd. Following an investigation by the publisher, all parties have concluded that this article was accepted solely on the basis of a compromised peer review process. The editors have therefore decided to retract the article. The authors did not respond to our notice regarding the retraction.

